# Decoding the genetic program of micronucleus formation: linking chromosomal instability to human disease

**DOI:** 10.1038/s41392-024-01869-2

**Published:** 2024-06-14

**Authors:** Jörg Fahrer

**Affiliations:** Division of Food Chemistry and Toxicology, Department of Chemistry, RPTU Kaiserslautern-Landau, 67663 Kaiserslautern, Germany

**Keywords:** Cancer genetics, Genomic instability

A recent study by Adams and colleagues published in *Nature* unveiled more than one hundred genes that are involved in micronucleus formation,^1^ which is a biomarker for genomic instability and associated with aging, cancer, and other disorders.^2^ As an outstanding achievement, the authors identified *DSCC1* (DNA Replication And Sister Chromatid Cohesion 1) as a critical gene protecting against early developmental defects, genomic instability, reduced proliferative capacity, cohesinopathy-related phenotypes, and tumor development in vivo.^1^

Micronuclei (MN) arise from chromosome mis-segregation, which results in the formation of chromosome fragments or intact chromosomes outside the nucleus after completion of mitosis.^3^ The micronucleated DNA, which resides in the cytoplasm, is surrounded by a nuclear envelope (NE) and associated with histones as well as nuclear proteins. Rupture of the NE promotes chromothripsis, i.e., a shattering of chromosomes followed by complex rearrangements due to the ligation of chromosome fragments, which drives chromosome hypermutation.^2,3^ Furthermore, a collapsed NE exposes the micronucleated DNA to cytosolic DNA-sensors of the innate immune system, resulting in activation of the cyclic GMP-AMP synthase (cGAS)-Stimulator of interferon genes (STING) pathway and inflammatory signaling. On these grounds, MN are not only mere biomarkers for cancer, but are also involved in cancer progression.^2,4^

**Fig. 1:**
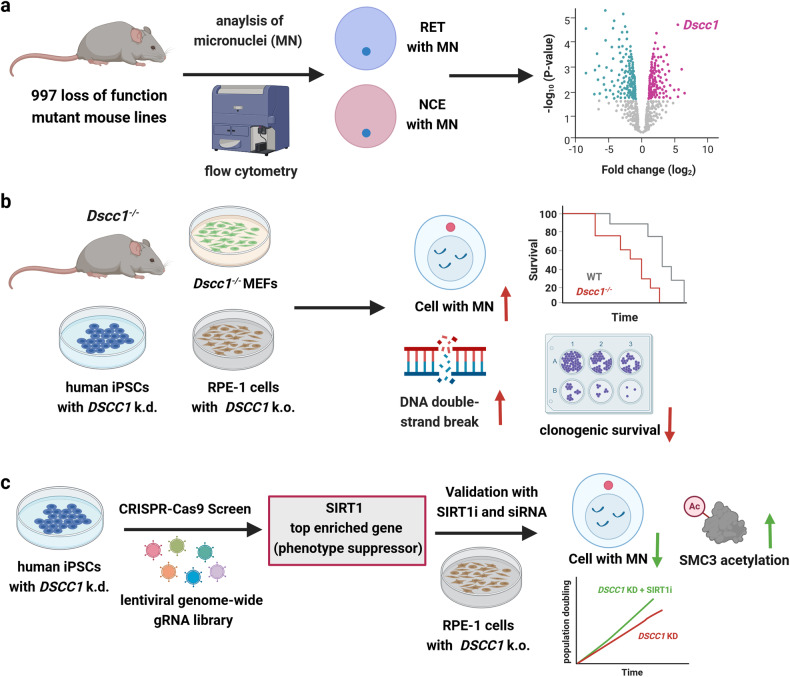
Identification of critical genes that control micronucleus formation in vivo and role of *DSCC1*. **a** Flow cytometry-based screening of 997 loss of function mouse lines for micronucleus (MN) formation in reticulocytes (RET) and normochromatic erythyrocytes (NCE). Loss of *DSSC1* caused the most significant increase in MN levels. **b** Knockout or knockdown of *DSCC1* in mouse and human models resulted in increased levels of genomic instability (e.g., γH2AX and MN formation), impaired cell growth, reduced survival with shorter tumor latency and phenotypes indicative of human cohesinopathies. **c** Identification of sirtuin 1 (SIRT1) as suppressor of *DSCC1* loss. Genetic abrogation of *SIRT1* or pharmacological inhibition (SIRT1i) rescued the phenotype associated with *DSCC1* loss, i.e., reduced the MN levels, promoted cell growth, and restored SMC3 acetylation. Created with BioRender.com

However, the genetic factors that control MN formation have been largely unknown. To address this knowledge gap, Adams and colleagues studied almost 1000 loss-of-function mutant mouse lines and assessed spontaneous MN formation in red blood cells. During erythrocyte maturation, normoblasts as precursor cells expel their nucleus, giving rise to reticulocytes (RET) and mature normochromatic erythyrocytes (NCE). MN formed in the precursor cells are retained in RET and NCE, which can be detected by propidium iodide DNA staining after RNA digestion (Fig. 1a). The used flow cytometry-based method was developed by members of the current team, allowing for high-throughput analysis of MN formation in a fast and sensitive manner. Using this key approach, 71 genes were identified to increase MN formation (+MN) if disrupted. Among those, several genes with known function in chromosome segregation, DNA damage response (DDR), and chromothripsis were found. On the other hand, 74 genes caused a phenotype with decreased MN formation (-MN) if disrupted, which is unique and has not been reported before. In order to validate these findings, the authors used CRISPR-Cas9 genome editing to generate human CHP-212 neuroblastoma cells deficient for selected genes. The cells were then exposed to hydroxyurea to moderately induce MN formation. Using this setup, knockout of *DSCC1* doubled the MN frequency to 10% as compared to wildtype cells (5%), whereas e.g., knockout of *DUSP7* (dual specificity phosphatase 7) decreased the MN frequency to 2%, being in line with the in vivo findings. Furthermore, the authors performed a phenotypic analysis of the mutant mouse lines (+MN and –MN), revealing that both show phenotypes such as increased mortality, growth retardation, dysfunction of the immune system and skeleton, as well as metabolic and neurological disorders, which are indicative of human cohesinopathies. These are disorders caused by mutations in the cohesin complex or its regulators. One prominent example is Cornelia de Lange syndrome, with mental and growth retardations as main phenotypes.

The authors then focused on *Dscc1*, whose loss-of-function caused the most significant increase in MN as compared to WT mice (Fig. 1a). *Dssc1*-mutant mice showed early developmental defects, particularly vascular anomalies in the liver and heart, while the surviving mice displayed skeletal abnormalities, increased body weight and bone mineral content, as well as reduced fertility, pathologies reminiscent of patients with cohesinopathies. Furthermore, experiments in *Dscc1*^*-/-*^ mouse embryonic fibroblasts revealed slower proliferation, increased levels of DNA damage, and chromosomal abnormalities. Consistent with these findings, *Dscc1*^*-/-*^ mice were prone to tumorigenesis in long-term studies and displayed reduced tumor-free survival. In order to confirm these results in a human model system, the authors generated induced pluripotent stem cells (iPS) with knockdown of *DSCC1*. Loss of *DSCC1* increased MN formation, reduced proliferation, and impaired sister chromatid cohesion. This comprehensive set of experiments with mouse and human models convincingly demonstrates that *DSCC1* is critical for genome stability and a tumor suppressor (Fig. 1b).

Next, the authors wanted to identify genes that interact with *DSCC1* loss. Their genome-wide CRISPR-Cas9 screen in human iPS cells with *DSCC1* knockdown yielded four genes that partially rescued the proliferation defect upon their genetic disruption, i.e., those are so-called phenotype suppressors. On the other hand, five genes were discovered that further decreased cell proliferation upon genetic disruption, which act as so-called phenotype enhancers. The four top suppressor genes were further validated in hTERT-immortalized retinal pigment epithelial (RPE) cells with a conditional *DSCC1* knockout, revealing an increased cell viability upon siRNA-mediated depletion of the four genes. One of those hits was *SIRT1 (sirtuin 1)*, which encodes a NAD^+^-dependent protein deacetylase. Typical substrates of SIRT1 are histones and proteins of the DDR, such as p53 and PARP-1. The genetic interaction between *DSCC1* and *SIRT1* was confirmed in human embryonic kidney (HEK) cells with *SIRT1* k.o. together with siRNA-mediated depletion of DSCC1. As another approach, the authors used a pharmacological SIRT1 inhibitor (SIRT1i) termed selisistat. SIRT1i treatment of both human iPS cells with *DSCC1* knockdown and hTERT-RPE-1 with conditional *DSCC1* knockout increased cell viability and reduced MN formation, which is consistent with the findings obtained by the genetic approaches (Fig. 1c).

To detail the underlying mechanism, the authors analyzed acetylation of SMC3 (Structural maintenance of chromosomes protein 3), which is a subunit of the cohesion complex that mediates sister chromate cohesion. Interestingly, a previous study showed that SMC3 acetylation on Lys105 was defective in *DSCC1*-deficient cells. In line with this report, the authors provided evidence that conditional *DSCC1* k.o. in RPE-1 cells reduced SMC3 acetylation and, at the same time, increased γH2AX and p53 levels. An intriguing finding was that SIRT1i not only restored SMC3 acetylation, but also decreased γH2AX and p53 to basal levels (Fig. 1c). Finally, the authors addressed the question whether SMC3 is a direct substrate of SIRT1-catalyzed deacetylation using immunoprecipitated proteins (SMC3, p53) and recombinant SIRT1. However, SIRT1 was not able to deacetylate SMC3, whereas it efficiently deacetylated p53. The exact mechanism by which SIRT1 inhibition restores SMC3 acetylation thus deserves further attention.

Altogether, Adams and colleagues identified more than 100 genetic factors, which govern MN formation in vivo. These groundbreaking findings will help to elucidate the mechanisms underlying MN formation and to clarify the causative role of MN in human disease, such as cancer, neurological disorders, and inflammatory diseases. These efforts are supported by a recent study on the proteomic landscape of MN following genotoxic stress.^5^ It would be very exciting to transfer this methodology to MN formed spontaneously in cells or mice defective for the genes identified in the landmark study by Adams and colleagues, such as *DSCC1*. Altogether, this expanding knowledge on MN should pave the way to develop new strategies for the treatment of human cohesinopathies and cancers with chromosomal instability.
